# *PPP3R1* Promotes MSCs Senescence by Inducing Plasma Membrane Depolarization and Increasing Ca^2+^ Influx

**DOI:** 10.3390/ijms24054421

**Published:** 2023-02-23

**Authors:** Molin Li, Weimin Gong, Jie Chen, Yining Zhang, Yufei Ma, Xiaolin Tu

**Affiliations:** Laboratory of Skeletal Development and Regeneration, Institute of Life Sciences, Chongqing Medical University, Chongqing 400016, China

**Keywords:** BMSC, aging, PPP3R1, Ca^2+^, membrane potential

## Abstract

Aging of mesenchymal stem cells(MSCs) has been widely reported to be strongly associated with aging-related diseases, including osteoporosis (OP). In particular, the beneficial functions of mesenchymal stem cells decline with age, limiting their therapeutic efficacy in age-related bone loss diseases. Therefore, how to improve mesenchymal stem cell aging to treat age-related bone loss is the current research focus. However, the underlying mechanism remains unclear. In this study, protein phosphatase 3, regulatory subunit B, alpha isoform, calcineurin B, type I (*PPP3R1*) was found to accelerate the senescence of mesenchymal stem cells, resulting in reduced osteogenic differentiation and enhanced adipogenic differentiation in vitro. Mechanistically, *PPP3R1* induces changes in membrane potential to promote cellular senescence by polarizing to depolarizing, increasing Ca^2+^ influx and activating downstream NFAT/ATF3/p53 signaling. In conclusion, the results identify a novel pathway of mesenchymal stem cell aging that may lead to novel therapeutic approaches for age-related bone loss.

## 1. Introduction

Age-related bone loss, or osteoporosis, is prevalent in animals and humans, and maintenance of skeletal homeostasis throughout life depends on the process of skeletal remodeling, which continuously replaces aged and damaged bones with new bones to maintain skeletal strength and elasticity. Recent studies suggested that senescent cells play a causal role in bone remodeling and the formation–resorption transition in age-related bone loss [1]. Multiple cell types in the skeletal microenvironment senesce with age [2].

Bone marrow mesenchymal stem cells (BMSCs) are multipotent progenitor cells with regenerative potential in various tissues [3]. Bone marrow stromal cell function dramatically declines with age [3,4]. Cellular senescence leads to a stable arrest of cell proliferation [5] and may affect aging BMSC function through intrinsic and extrinsic mechanisms. Aging is a complex, gradual, and inevitable physiological process, accompanied by the accumulation of damaged macromolecules, leading to organ dysfunction and the senescence of MSCs, with the character of significantly impaired biological properties of MSCs [6,7,8]. Several methods have been tried to modify the senescence of MSCs. For example, BMP2 as well as TGFβ signaling play an important role in the aging process of MSCs [9,10], but the mechanism of MSCs aging remains to be investigated.

Calcium homeostasis is undoubtedly an important mechanism of action in various types of studies on cellular and organ aging [11,12,13,14,15]. Calcineurin is a Ca^2+^-dependent phosphatase that is expressed in many cell types, including osteoblasts. It dephosphorylates nuclear factors of activated T cell (NFAT) proteins, which are first involved in T cell activation by controlling interleukin-2 expression [16]. However, calcineurin–NFAT signaling is also effective in osteoblasts [16]. Calcineurin inhibitors such as cyclosporine A (CsA) or tacrolimus (FK-506) are widely used as immunosuppressive drugs to treat patients after organ transplantation or with severe autoimmune diseases [17,18].

Cells have a transmembrane potential, which is maintained by the balance between ions on both sides of the plasma membrane. Although the underlying mechanism is unknown, alterations in membrane potential have been found to be associated with senescence in fibroblasts and epithelial cells [19].

Since membrane potential as well as calcineurin have been reported to be associated with cellular senescence, we hypothesized that membrane depolarization may play a role in Ca^2+^-dependent calcineurin PPP3R1 (protein phosphatase 3, regulatory subunit B, α isoform; also known as CNB1) -regulated stromal stem cell senescence and further revealed specific mechanisms. The discovery of this mechanism will provide new clues for a novel electrophysiological pathway to control aging and bone aging in stromal stem cells.

## 2. Results

### 2.1. PPP3R1 Is Activated in Senescent BMSCs

**Figure 1:**
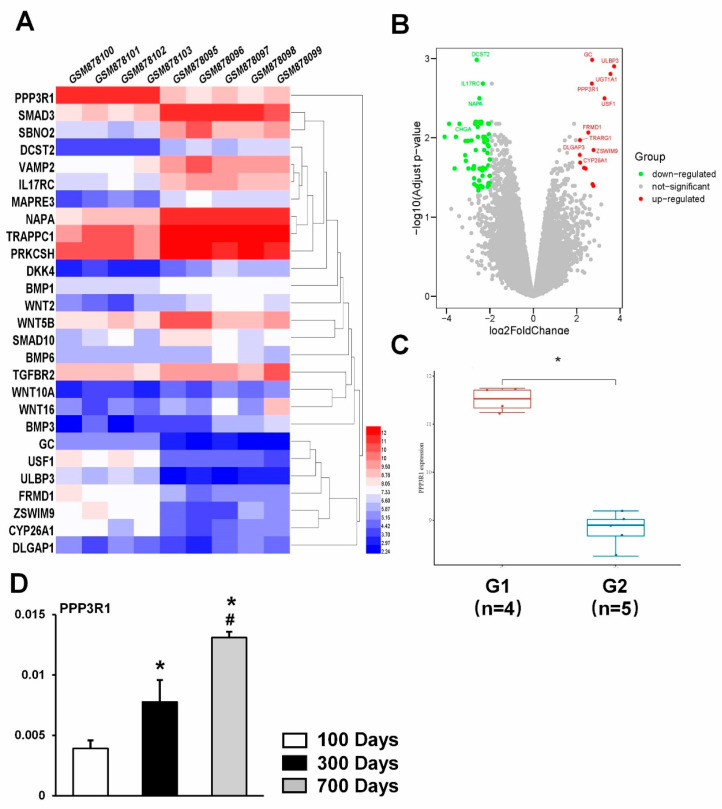
PPP3R1 is activated in senescent BMSCs. (**A**) The heatmap of the differential gene expression; different colors represent the trend of gene expression in different tissues. (**B**) Volcano plot: The volcano plot was constructed using the fold change values and P-adjust. Red dots indicate upregulated genes. Green dots indicate downregulated genes. (**C**) The expression distribution of PPP3R1 gene in different tissue. The abscissa represents different groups of samples, and the ordinate represents the expression distribution of the gene; different colors represent different groups. (**D**) Expression levels of PPP3R1 in BMSCs from mice of different ages. * *p* < 0.05. *^#^ p* < 0.05 vs. 300 Days Group.

By GEO database analysis, PPP3R1 mRNA expression was significantly higher in samples from four older age groups in nine sets of samples from dataset GSE35955 (Figure 1A). Volcano plots showed the number of differentially expressed genes identified from each dataset. Similarly, PPP3R1 mRNA expression was found to be significantly higher in samples from the older age group (Figure 1B). Expression of PPP3R1 was elevated ≥2.7 in the older group compared with the younger group (Figure 1C). In the meantime, to determine the role of PPP3R1 in BMSC cell senescence, we examined the levels of PPP3R1 in BMSCs from mice of different ages and found that the expression of PPP3R1 in the BMSCs increased step by step with mouse age (Figure 1D).

### 2.2. Establishment of Senescent Stromal Stem Cell Lines

**Figure 2:**
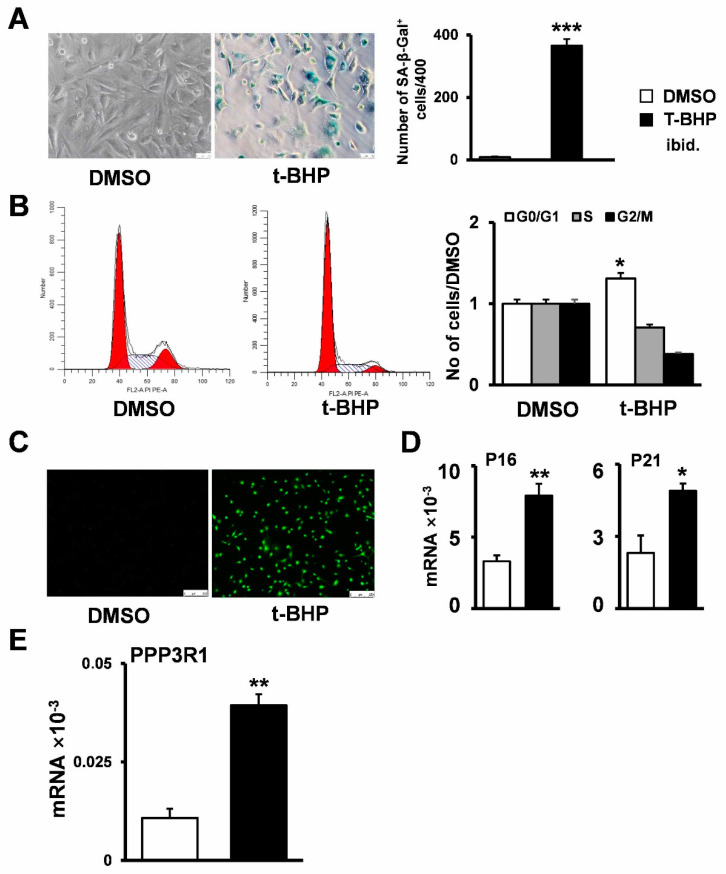
Tert-butyl hydrogen peroxide induced senescence in C3H10T1/2 cells. Cell senescence status was evaluated by (**A**) cell senescence β-galactosidase staining, (**B**) cell cycle detection, (**C**) ROS staining, and (**D**) qPCR after tert-butyl hydroperoxide treatment of cells for 6 h. (**E**) The mRNA expression of PPP3R1 was detected by qPCR. * *p* < 0.05. ** *p* < 0.01. *** *p* < 0.001. (**B**) red areas: G0/G1 and G2 phase; (**B**) shadow areas: S phase. (Scale bars: (**A**) 20×, (**C**) 20×).

To investigate the expression and role of PPP3R1 in the senescence of MSCs in vitro, C3H10T1/2 cells were treated with tert-butyl hydroperoxide (t-BHP) to obtain senescent MSC cell lines [20]. As expected, tert-butyl hydroperoxide treatment increased SA-β-Gal activity in C3H10T1/2 cells (Figure 2A), resulted in proliferation arrest (Figure 2B), increased cellular reactive oxygen species production (Figure 2C), and induced increased expression of the senescence markers p16 and p21 (Figure 2D). In parallel, PPP3R1 mRNA expression in cells was detected, and tert-butyl hydroperoxide treatment was found to increase PPP3R1 expression in C3H10T1/2 cells (Figure 2E). Overall, we successfully generated senescent stromal stem cell lines using the tert-butyl hydroperoxide treatment of C3H10T1/2 cells and found increased PPP3R1 expression.

### 2.3. PPP3R1 Inhibition Enables C3H10T1/2 Escape from Senescence

**Figure 3:**
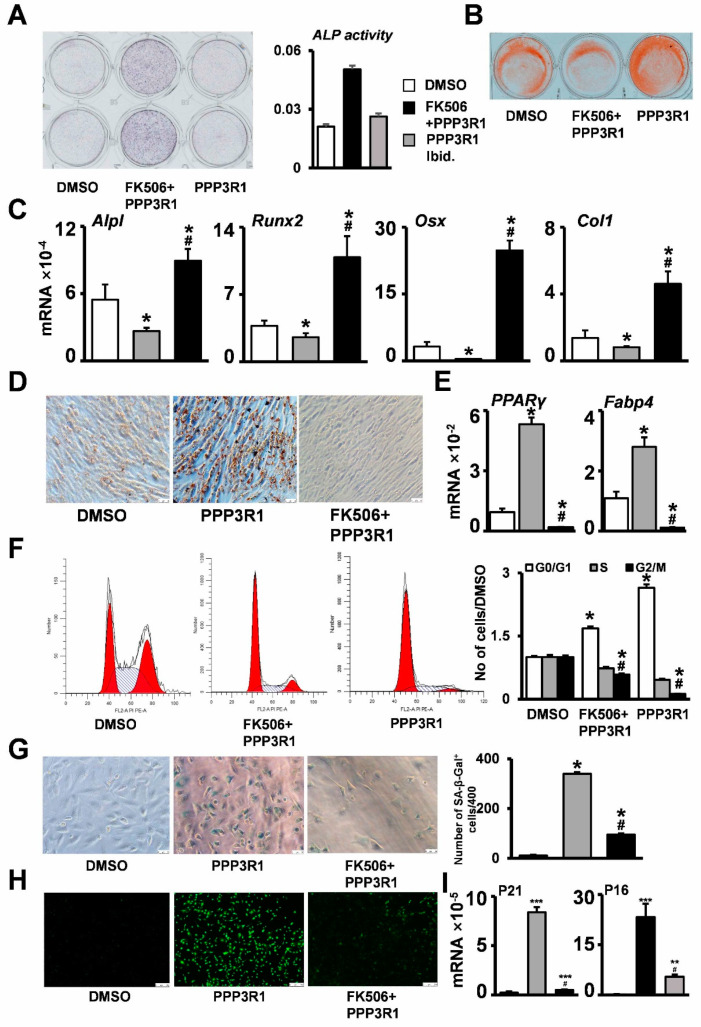
PPP3R1 inhibition enables C3H10T1/2 escape from senescence. Osteogenic differentiation ability was evaluated by (**A**) ALP staining, (**B**) alizarin red staining, and (**C**) qPCR in C3H10T1/2 cells treated with PPP3R1 recombinant protein as well as FK506. Changes in adipogenic differentiation ability were detected by (**D**) oil red O staining as well as (**E**) qPCR. Changes in cellular senescence status were assessed by (**F**) cell cycle assays, (**G**) β-galactosidase staining, (**H**) ROS staining, and (**I**) qPCR assays. *^#^ *p* < 0.05. ** *p* < 0.01. *** *p* < 0.001. (**F**) red areas: G0/G1 and G2 phase; (**F**) shadow areas: S phase (Scale bars: (**A**) 100 μm, (**B**) 100 μm, (**D**) 25 μm, (**G**) 50 μm, (**H**) 250 μm).

To better characterize the role of PPP3R1 in stromal stem cell senescence, we generated stromal stem cell lines in which PPP3R1 was activated as well as inhibited by treating C3H10T1/2 cells with PPP3R1 recombinant protein and with FK506. Markers of senescence in stromal stem cells were mainly proliferation arrest, reduced osteogenic differentiation ability, and enhanced adipogenic differentiation ability. We also found that C3H10T1/2 cells had reduced osteogenic differentiation ability (Figure 3A–C), substantially enhanced adipogenic differentiation ability (Figure 3D,E), and showed proliferation arrest (Figure 3F) when PPP3R1 was activated. At the same time, C3H10T1/2 cells showed enhanced activity of SA-β-Gal (Figure 3G), increased cellular reactive oxygen species production (Figure 3H), and greatly enhanced expression of the senescence marker P16P21 (Figure 3I). However, the above results were reversed after PPP3R1 was inhibited using FK506 (Figure 3A–I). On the basis of these results, we conclude that PPP3R1 inhibition enables mesenchymal stem cells to escape senescence.

### 2.4. Plasma Membrane Depolarization Induces C3H10T1/2 Cell Senescence

**Figure 4:**
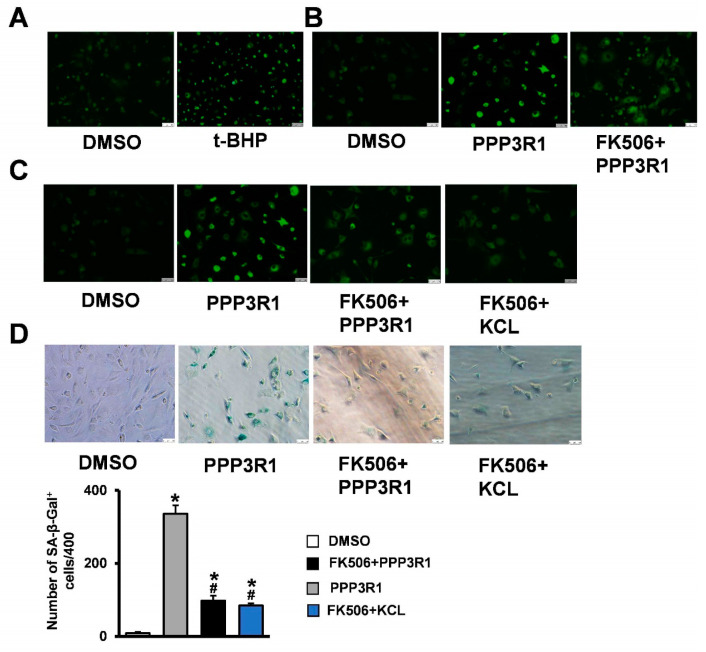
Plasma membrane depolarization induces C3H10T1/2 cell senescence. (**A**) Detection of plasma membrane potential changes in senescent cells by DiBAC4 fluorescent dye staining. After the cells were treated with PPP3R1 recombinant protein, FK506 and KCL, the changes of plasma membrane potential were detected by (**B**,**C**) DiBAC4 fluorescent dye staining, and cell senescence was detected by (**D**) β-galactosidase staining. *^#^ *p* < 0.05. (Scale bars: (**A**) 100 μm, (**B**) 100 μm, (**C**) 100 μm, (**D**) 50 μm).

Then, we tried to preliminarily clarify the mechanisms responsible for the regulation of MSC senescence by PPP3R1. Recently, membrane depolarization has been reported to be associated with cell senescence [19,21]. We therefore investigated whether changes in plasma membrane potential might play a role in PPP3R1-regulated senescence in C3H10T1/2 cells. To measure their relative plasma membrane potential, the fluorescent dye DiBAC4 was used to incubate senescent C3H10T1/2 cells with increased uptake in cells with depolarized plasma membranes (decreased uptake in hyperpolarized cells). As mentioned above, C3H10T1/2 cells presenting senescence showed higher DiBAC4 fluorescence intensity, whereas control cells showed lower plasma membrane depolarization (Figure 4A). Meanwhile, PPP3R1-activated C3H10T1/2 cells showed high DiBAC4 fluorescence intensity, and C3H10T1/2 cells in PPP3R1 inhibited by FK506 showed low DiBAC4 fluorescence intensity (Figure 4B). We further treated FK506-treated C3H10T1/2 cells with potassium chloride, a well-established plasma membrane depolarizer. Forced depolarization in C3H10T1/2 cells abrogated the favorable effect of PPP3R1 inactivation on cellular senescence (Figure 4C,D). These results suggest that PPP3R1 regulates senescence in stromal stem cells by controlling plasma membrane potential.

### 2.5. Plasma Membrane Depolarization Increases Ca^2+^ Influx and Activates NFAT/ATF3/p53 Signaling, Thereby Inducing MSC Senescence

**Figure 5:**
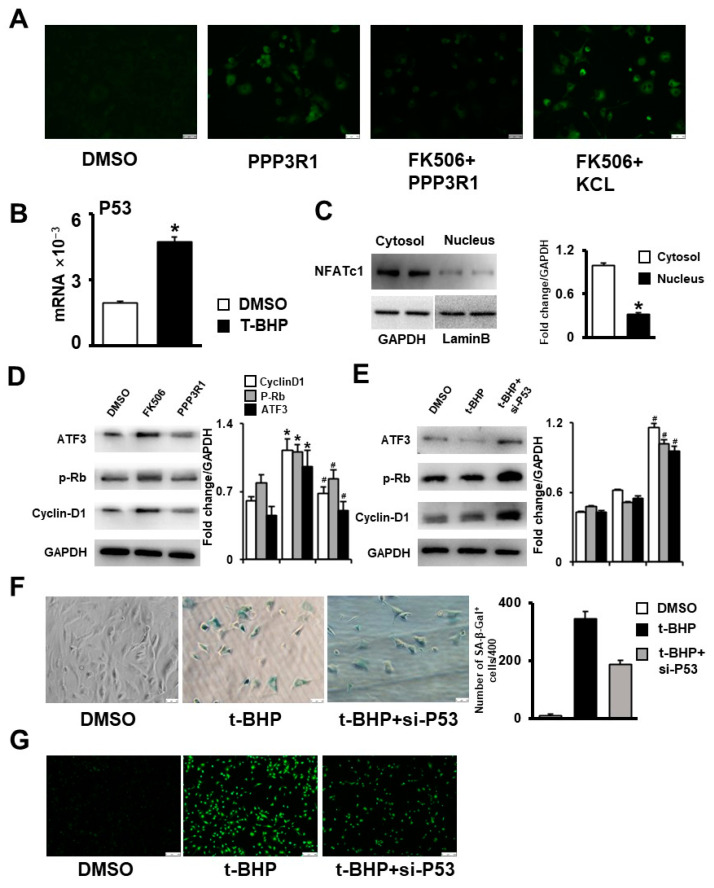
Plasma membrane depolarization increases Ca^2+^ influx and activates NFAT/ATF3/p53 signaling, thereby inducing MSC senescence. After the cells were treated with PPP3R1 recombinant protein, FK506 and forced depolarization agent KCL, the changes of calcium ions in the cells were detected by (**A**) calcium ion fluorescent probe, and the expression of P53 in senescent cells was detected by (**B**) qPCR. (**C**) WB was used to detect the protein expression of NFATc1 in the cytoplasm and nucleus of senescent cells. (**D**,**E**) WB was used to detect the protein expression of ATF3, p-Rb, and Cyclin-D1 in different groups. (**F**) β-galactosidase staining was used to detect cell senescence after interfering with P53 expression. (**G**) ROS staining was used to detect the changes of reactive oxygen species after interfering with P53 expression. *^#^ *p* < 0.05 (Scale bars: (**A**) 100 μm, (**F**) 50 μm, (**G**) 250 μm).

Finally, we investigated the mechanism by which plasma membrane depolarization promotes senescence in C3H10T1/2 cells. Depolarization of the plasma membrane has been shown to activate voltage-gated calcium channels and increase intracellular calcium [22], whereas this increased calcium promotes cellular senescence [23,24]. Our study found decreased calcium ion levels in senescent C3H10T1/2 cells but increased depolarization induced by potassium chloride (Figure 5A).

The increase in intracellular calcium induced cellular senescence by triggering NFAT dephosphorylation and translocating it to the nucleus [25]. NFATc1 inhibits the expression of ATF3, which in turn increases the expression of P53 and other aging-related markers [26]. P53 induces transcription of the Cyclin-dependent kinase inhibitor p21. In turn, p21 inhibits the activity of CDK4/6, which leads to a decrease in Cyclin-D1 levels, a decrease in the degree of Rb phosphorylation, and cell cycle exit [27]. Consistent with these findings, we observed increased P53 expression in senescent C3H10T1/2 cells (Figure 5B), and nuclear translocation of NFATc1 was promoted (Figure 5C), while ATF3 expression was decreased in PPP3R1-activated C3H10T1/2 cells, which further increased P53 expression in cells and decreased Cyclin-D1 levels and Rb phosphorylation in cells (Figure 5D). Meanwhile, after interfering with P53 expression, senescent C3H10T1/2 cells showed increased Cyclin-D1 levels and Rb phosphorylation levels (Figure 5E), and the senescent phenotype was alleviated (Figure 5F,G). These results suggest that plasma membrane depolarization leads to stromal stem cell senescence by increasing Ca^2+^ influx and activating the NFAT/ATF3/p53 signaling pathway.

## 3. Discussion

Mesenchymal stem cells are characterized by their ability to proliferate and maintain an undifferentiated state as well as their ability to differentiate into multiple cell lineages. Bone marrow mesenchymal stem cells (MSCs) are generally considered to be the best source of MSCs. However, the number and differentiation potential of mesenchymal stem cells showed an age-related decline. Understanding the mechanisms of pre-aging of stromal stem cells is critical for developing therapeutic interventions for age-related bone loss. In this study, PPP3R1 was identified as a regulator of senescence in stromal stem cells using tert-butyl hydroperoxide co-treatment with C3H10T1/2 cells, and the further results showed that *PPP3R1* promotes plasma membrane depolarization in senescent stromal cells, which promotes cellular senescence by increasing Ca^2+^ influx and activating downstream NFAT/ATF3/p53 signaling. Therefore, *PPP3R1* might be a novel regulator during aging in stromal stem cells.

Calcineurin has undoubtedly been an important research direction in previous studies on aging [26,28,29,30]. Calcineurin (protein phosphatase 3, PPP3) is a widely expressed calcium-sensitive serine-threonine phosphatase consisting of the 61 kD calmodulin-binding catalytic subunit A (gene name, *PPP3C*) and the 19 kD Ca^2+^-binding regulatory subunit B (gene name, *PPP3R*) [31]. Three genes encode catalytic subunit A: *PPP3CA* (isoform a), *PPP3CB* (isoform b), or *PPP3CC* (isoform g), and *PPP3CA* and *PPP3C* show partially overlapping expression patterns and functions [32]. For regulatory subunit B, two genes (*PPP3R1* and *PPP3R2*) have been described, but *PPP3R2* expression appears to be confined to the testis [33]. Calcineurin is activated by intracellular calcium influx and represents a critical signaling node that transmits environmental stimuli into adaptive responses in multiple tissues and organs. In this study, we found that there was a large difference in *PPP3R1* expression in mouse BMSCs at different ages, and further studies revealed that activation of PPP3R1 accelerated the aging of stromal stem cells and altered the differentiation ability of stromal stem cells into bone and adipocyte lineages, while the opposite was true when stromal stem cells were treated with FK506, a calcineurin inhibitor.

Cells have a transmembrane potential, which is maintained by a balance between ions on either side of the plasma membrane. Although studies of membrane potential have focused on excited cells, recent studies have shown that dynamic membrane potential is also present in most non-excitatory cells, but its mechanism of action is unknown [34]. Changes in plasma membrane potential during cellular senescence were first reported by Lallet-Daher et al. [35]. Since then, senescence in epithelial cells [19] and fibroblasts [21] has also been attributed to changes in membrane potential in several studies, although the specific mechanisms are unknown. In the present study, we show that plasma membrane depolarization induces stromal stem cell senescence by increasing Ca^2+^ influx and activating downstream NFAT/ATF3/p53 signaling.

Voltage-gated calcium (Ca^2+^) channels (VGCCs) are critical sensors for the conversion of membrane potential changes into intracellular Ca^2+^ transients [36]. In parallel, Cav1.2 has been found to be constitutively expressed by preosteoblasts and may mediate Ca^2+^ influx in response to depolarization [37]. In addition, Fei et al. found that Cav1.2 itself promotes osteogenesis of bone marrow-derived mesenchymal stem cells, and upregulation of Cav1.2 expression alleviates osteoporosis in prematurely aging mice [38]. This suggests that a similar mechanism can also exist in our study, which requires further study and confirmation at a later stage.

In conclusion, we reveal the mechanism by which *PPP3R1*/plasma membrane depolarization induces senescence in stromal stem cells, including *PPP3R1*-promoting plasma membrane depolarization which produces membrane potential changes as well as calcium influx, plasma membrane depolarization, and calcium involvement in the regulation of the NFAT/ATF3/P53 pathway. Thus, this work provides novel insights into the involvement of ion channels and plasma membrane potential in controlling stromal stem cell senescence. Pharmaceutical studies of related pathways and drugs may lead to new potential treatments for the use of stem cells during age-related bone loss.

## 4. Materials and Methods

### 4.1. Microarray Analysis

All microarray data were downloaded from the GEO database (http://www.ncbi.nih.gov/geo;GSE35955; accessed on 12 April 2022). The raw data were downloaded as MINiML files which contain the data for all platforms, samples, and GSE records of the GSE. The extracted data were normalized by log2 transformation. The microarray data were normalized by the normalize quantiles function of the preprocessCore package in R software (version 3.4.1). Probes were converted to gene symbols according to the annotation information of the normalized data in the platform. Probes matching multiple genes were removed from these datasets. The average expression value of gene measured by multiple probes was calculated as the final expression value. In cases of the same dataset and platform but in different batches, we used the removeBatchEffect function of the limma package in the R software to remove batch effects. In cases of different datasets or in the same dataset but in different platforms, extracting multiple datasets with common gene symbols, and marking different datasets or different platforms as different batches, we used the removeBatchEffect function of the limma package in the R software to remove batch effects. The result of the data preprocessing was assessmented by boxplot.

### 4.2. Cell Culture and Treatment

Mouse embryo-derived mesenchymal stem cells (C3H10T1/2) were purchased from the American Type Culture Collection, while we also isolated primary BMSCs from the long bones of mice [39]. Cells were maintained in α-MEM medium (αMEM, Corning, New York, NY, USA) containing 10% FBS (Gibco, New York, NY, USA), 100 U mL^−1^ penicillin/streptomycin, and 5% CO_2_ at 37 °C. We used tert-butyl hydroperoxide (t-BHP) (Sigma-Aldrich, Gaithersburg, MD, USA, 100 µmol·L^−1^) treatment to induce cellular senescence, and C3H10T1/2 cells were continuously cultured with tert-butyl hydroperoxide for 6 h. To induce osteogenic differentiation, the C3H10T1/2 cells were seeded at a density of 1.5 × 10^5^ cells/well (six-well plates) and incubated with osteoblast differentiation medium containing β-glycerophosphate (Sigma-Aldrich, 5 mmol/L) and ascorbic acid (Sigma-Aldrich, 50 g/mL). To alter PPP3R1 expression, the C3H10T1/2 cells were cultured with PPP3R1 murine recombinant protein (CUSABIO, Houston, TX, USA, CSB-EP737231MO) as well as FK506 (MedChemExpress, Monmouth Junction, NJ, USA, HY-13756).

### 4.3. Cell Staining

For SA-β-galactosidase staining, cells were washed with PBS, fixed with 4% paraformaldehyde in PBS at room temperature for 20 min and incubated with reagents from a senescence-associated β-galactosidase staining kit (Beyotime Institute of Biotechnology, Shanghai, China, #C0602) according to the manufacturer’s suggestions.

For alkaline phosphatase staining: Treated C3H10T1/2 cells were washed with a phosphate buffer, fixed in 4% paraformaldehyde for 30 min at room temperature, and stained with an alkaline phosphatase staining kit (Beyotime Institute of Biotech, Shanghai, China) for 1 h at room temperature in the dark.

For alizarin red staining, cells were fixed with paraformaldehyde for 30 min, incubated with 1% alizarin red for 30 min at room temperature and washed with PBS to remove the excess dye.

### 4.4. Western Blotting

Protein samples were extracted using a cell lysis buffer (P0013, Beyotime, Shanghai, China) supplemented with proteinase (04693159001, Roche, Basel, Switzerland) and phosphatase inhibitors (4906837001, Roche, Basel, Switzerland). Then, a 1/5 volume of loading buffer was added into the cell lysis at 100 °C for 10 min. Protein samples were separated by sodium dodecyl sulfate polyacrylamide gel electrophoresis and then transferred onto polyvinylidene fluoride membranes. Blots were blocked in 5% bovine serum albumin for 2 h at 25 °C, and then the membrane was incubated with specific antibodies against NFATc1 (Cell Signaling Technology, Boston, MA, USA, 1:1000, #8032), p-Rb (Bioss, Beijing, China, 1:1000, bsm-52197R), ATF3 (Abbkine, Beijing, China, 1:1000, #Abp55330) and Cyclin-D1 (Bioss, Beijing, China, 1:1000, bs-0623R) at 4 ℃ overnight. Blots were incubated with a secondary antibody conjugated to horseradish peroxidase (diluted 1:5000) for 2 h at 25 °C. Finally, each membrane was exposed to ECL (SQ202, Epizyme, Shanghai, China).

### 4.5. RNA Extraction and Gene Expression Analysis

The total RNA of cells was extracted using the TRIzol reagent (15596026, Invitrogen, Shanghai, China). Following evaluation of the RNA concentration, the DNA group was removed and cDNA was obtained through reverse transcription using a reverse transcription kit (RR047A, Takara, Beijing, China) according to the manufacturer’s instructions. The IQ SYBR Green Supermix (RR820, Takara, Beijing, China) was used to conduct 3 biological duplications of real-time quantitative PCR on an iCycler real-time detection system (CFX Connect Optics Module, Bio-Rad, Hercules, CA, USA). The corresponding gene expression level was normalized to that of GAPDH from the same samples. Table 1 provides the primer sequences.

### 4.6. Cell Cycle Assay

To assess the cell cycle, we seeded 3 × 10^5^ treated cells into six-well plates and incubated them at 37 °C for 48 h. For the cell cycle analysis, cells were digested with trypsin (Hyclone, Logan, UT, USA), washed twice with phosphate-buffered saline (PBS), and fixed in 70% ethanol at 4 °C overnight. The cells were centrifuged at 500× *g* for 5 min, washed twice with cold PBS, and centrifuged. Cell cycle analysis was performed using fluorescence-activated cell sorting after the digested cells were treated with RNase A (0.1 mg/mL) and stained with propidium iodide (0.05 mg/mL; 4A Biotech, Beijing, China) for 30 min at 37 °C.

### 4.7. Plasma Membrane Potential Measurement

Cultured cells were washed with a HEPES buffer (pH 7.4; 20 mM HEPES, 120 mM NaCl, 2 mM KCl, 2 mM CaCl_2_, 1 mM MgCl_2_, 5 mM glucose) and incubated with 5 μmol·L^−1^ membrane voltage-reporter dye DiBAC4 (Molecular Probes) for 1 h at 37 °C. Then, the cells were observed and photographed with an inverted fluorescence microscope (Olympus, Tokyo, Japan). Fluorescence data were analyzed with Image-J software.

### 4.8. Cytosolic Calcium Level Measurements

Cytosolic calcium levels were detected with a Fura-2/AM (Invitrogen™, Shanghai, China, # F1221) according to the manufacturer’s suggested procedure. C3H10T1/2 cells were loaded with 5 μmol·L^−1^ Fura-2/AM in Hanks’ balanced salt solution (HBSS) for 1 h at 37 °C. After the cells were washed extensively with HBSS, cytosolic Ca^2+^ was measured with a calcium imaging system built on an inverted fluorescence microscope (Olympus IX51, Tokyo, Japan). Fluorescence images (filtered at 515 nm ± 25 nm) were captured with a CCD camera (CoolSNAP fx-M) and analyzed in MetaFluor software. Ca^2+^ levels are shown as the ratio of fluorescence intensity at 340 nm/fluorescence intensity at 380 nm (F340/F380). At least three independent experiments were performed for each condition.

### 4.9. Statistical Analysis

All results are presented as the mean ± SD. Curve analysis was performed in Prism (GraphPad). The data in each group were analyzed with an unpaired, two-tailed Student’s *t*-test. The significance threshold was set at *p* < 0.05.

## 5. Conclusions

In summary, this study found that PPP3R1 increases Ca^2+^ influx by polarization depolarization, activates downstream NFAT/ATF3/p53 signaling, induces membrane potential changes, promotes cell senescence, and leads to decreased osteogenic differentiation and enhanced adipogenic differentiation. Taken together, the results identify a novel mesenchymal stem cell senescence pathway that may lead to new treatments for age-related bone disease.

## Figures and Tables

**Table 1:** Sequences of primers used for qRT-PCR (mouse).

Primer	Forward	Reverse
GAPDH	GCACAGTCAAGGCCGAGAAT	GCCTTCTCCATGGTGGTGAA
PPP3R1	CCAACACGGTCCCCATTA	TTGGCAAAGCGGACTTTC
P16	CAAGAGCGGGGACATCAAGACATC	CACAAAGACCACCCAGCGGAAC
P21	TCCTGGTGATGTCCGACCTGTTC	ACGAAGTCAAAGTTCCACCGTTCTC
P53	ACCGCCGACCTATCCTTACCATC	GGCACAAACACGAACCTCAAAGC
Alp	ACACCAATGTAGCCAAGAATGTCA	GATTCGGGCAGCGGTTACT
Runx2	CCGGTCTCCTTCCAGGAT	GGGAACTGCTGTGGCTTC
Osx	CCCTTCTCAAGCACCAATGG	AAGGGTGGGTAGTCA TTTGCATA
Col1	GAGCGGAGAGTACTGGATCG	GCTTCTTTTCCTTGGGGTTC
PPARγ	AGCCCTTTACCACAGTTGATTTCTCC	GCAGGTTCTACTTTGATCGCACTTTG
FABP4	GACGACAGGAAGGTGAAGAGCATC	GGAAGTCACGCCTTTCATAACACATTC

## Data Availability

All data generated or analyzed during this study are included in this published article.
